# The complete mitochondrial genome of the symbiotic infaunal snapping shrimp *Leptalpheus forceps* (Decapoda, Alpheidae)

**DOI:** 10.1080/23802359.2019.1704650

**Published:** 2020-01-16

**Authors:** Justin A. Scioli, Sophie Plouviez, Darryl L. Felder

**Affiliations:** Department of Biology, University of Louisiana at Lafayette, Lafayette, LA, USA

**Keywords:** Alpheidae, *Leptalpheus*, mitogenome, mitochondrial genome, infauna

## Abstract

The snapping shrimp *Leptalpheus forceps* (family Alpheidae) has a unique natural history as an infaunal symbiont of larger burrowing crustaceans. The mitogenome of *L. forceps* was sequenced, the first for a symbiotic representative of the family and the first for a species outside of the genus *Alpheus*. The complete mitogenome was 15,463 bp in length and included 13 protein-coding genes, 12 tRNAs, and 2 rRNAs. The gene order matched all known alpheid mitogenomes. Similar to other caridean mitogenomes, the nucleotide composition was A + T biased (62%). A maximum-likelihood phylogenetic analysis of caridean mitogenomes strongly supported monophyly of the family Alpheidae.

Snapping shrimp (Decapoda: Alpheidae) include species that have evolved obligate and facultative symbioses with members of at least eight animal phyla (Bruce 1976; Anker 2001). The snapping shrimp *Leptalpheus forceps* is an obligate associate of burrowing ghost shrimps in the families Callianassidae and Upogebiidae, inhabiting the burrow of these hosts (Williams 1965; Dawson 1967; Saloman 1971; Felder and Rodrigues 1993). While molecular phylogenetic methods provide an opportunity to investigate the evolution of symbiotic lifestyles in Alpheidae, the only alpheid mitogenomes that have been completely sequenced to date are for free-living (nonsymbiotic) species. We sequenced the whole mitogenome of *L. forceps* to contribute to genetic information on symbiotic alpheid shrimps.

A specimen of *L. forceps* was collected with a hand-operated extraction corer (‘yabby pump’, Manning 1975) from a burrow of the ghost shrimp *Lepidophthalmus louisianensis* in Bay St. Louis, Mississippi, USA. The specimen was kept frozen until DNA extraction, after which it was preserved in 70% ethanol and cataloged in the University of Louisiana at Lafayette Zoological Collection under the accession no. ULLZ18053. We extracted genomic DNA from abdominal muscle tissue using the Qiagen DNeasy Blood and Tissue Kit (Cat. No. 69504) following the manufacturer’s instructions. We prepared genomic DNA library using the Nextera XT Library Preparation Kit (Illumina, Cat. No. FC-131-1024) following the manufacturer’s instructions. The library was sequenced (paired-end, 2 x 250 bp) on the Illumina MiSeq Nano v2 platform at the Clinical Genomics Center at the Oklahoma Medical Research Foundation. We obtained 767,201 paired-end reads with a total of 192 million bases. A partial mitogenome assembly was generated by mapping our reads against the *Alpheus japonicus* mitogenome in Geneious Prime ver. 2019.1.1 using blastn. We used a 672 bp putative *cox2* sequence from this partial assembly as a seed sequence to assemble the entire mitogenome using NovoPlasty ver. 2.7.2 (Dierckxsens et al. 2017) and recovered the mitogenome as a single contig. We annotated the mitochondrial genes using the MITOS pipeline (Bernt et al. 2013) and manually curated the MITOS output with alpheid mitogenomes. The complete mitogenome of *L. forceps* is available at Genbank under accession no. MN_732884.

The mitogenome of *L. forceps* was 15,463bp in length and contained the same set of 13 protein coding genes, 22 tRNAs, and 2 rRNAs as other caridean mitogenomes. The overall base composition of the mitogenome was estimated to be A = 33.8%, C = 24.9%, G = 13.1%, and T = 28.2%, with a high A + T content of 62%, which is within the range of A + T content of published alpheid mitogenomes. We conducted a maximum likelihood phylogenetic analysis using a concatenated alignment of the 13 protein-coding mitochondrial genes of *L. forceps* and 12 other caridean shrimps, as well as outgroups. Similar to previous studies, we recovered strong bootstrap support (100) for an Alpheidae + Palaemonidae clade as well as for the family Alpheidae, with *L. forceps* diverging from the four *Alpheus* mitogenomes (Figure 1). The sequencing of additional alpheid mitogenomes will likely reveal closer phylogenetic relationships to *Leptalpheus* than *Alpheus*, as these genera are considered only distantly related within Alpheidae.

**Figure 1.**
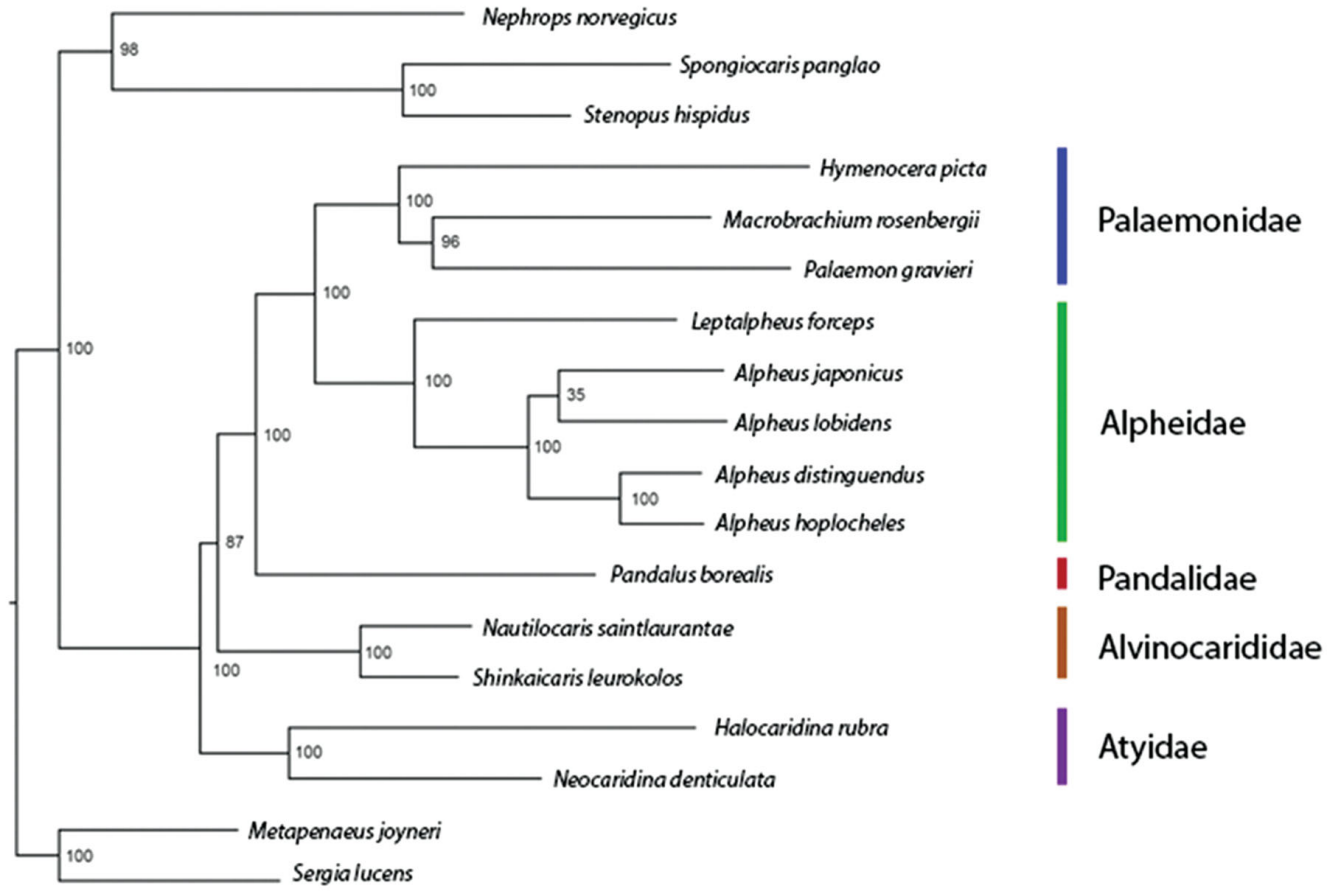
Maximum-likelihood phylogenetic tree of *Leptalpheus forceps* with 12 other caridean shrimp species, as well as dendrobranchiate, stenopodidean and astacidean outgroups. Values of internal nodes represent bootstrapping support (1000 iterations). The GenBank accession numbers of mitogenomes used in this analysis are the following: *Nephrops norvegicus* LN_681403, *Spongiocaris panglao* NC_038166, *Stenopus hispidus* NC_018097, *Hymenocera picta* NC_039631, *Macrobrachium rosenbergii* AY_659990, *Palaemon gravieri* KT_953323, *Leptalpheus forceps* MN_732884, *Alpheus japonicus* MG_787409, *A. lobidens* KP_276147, *A. distinguendus* GQ_892049, *A. hoplocheles* MG_873459, *Pandalus borealis* LC_341266, *Nautilocaris saintlaurantae* NC_021971, *Shinkaicaris leurokolos* NC_037487, *Halocaridina rubra* DQ_917432, *Neocaridina denticulata* NC_023823, *Metapenaeus joyneri* MH_939247, *Sergia lucens* LC_368254.
